# Compound Salt-Based Coagulants for Tofu Gel Production: Balancing Quality and Protein Digestibility

**DOI:** 10.3390/gels11070524

**Published:** 2025-07-06

**Authors:** Zhaolu Li, Sisi Zhang, Zihan Gao, Xinyue Guo, Ruohan Wang, Maoqiang Zheng, Guangliang Xing

**Affiliations:** School of Biology and Food Engineering, Suzhou University of Technology, Changshu 215500, China

**Keywords:** tofu, gel strength, in vitro digestion, protein bioavailability

## Abstract

Tofu quality is critically influenced by coagulants, though their impact on protein digestibility remains underexplored. This study aimed to investigate the effects of calcium sulfate (CaSO_4_), magnesium chloride (MgCl_2_), and their combination (CaSO_4_ + MgCl_2_) on the physicochemical properties and protein digestibility of tofu. Water-holding capacity, cooking loss, texture, protein composition, and protein digestibility were analyzed. The results showed that the CaSO_4_ + MgCl_2_ combination yielded a water-holding capacity of 99.16%, significantly higher than CaSO_4_ tofu (93.73%) and MgCl_2_ tofu (96.82%), while reducing cooking loss to 2.03% and yielding the highest hardness (897.27 g) and gumminess (765.72). Electrophoresis revealed distinct protein retention patterns, with MgCl_2_ (0.6% *w*/*v*) forming denser gels that minimized protein leakage into soy whey. During in vitro digestion, MgCl_2_-coagulated tofu exhibited superior soluble protein release (5.33 mg/mL after gastric digestion) and higher intestinal peptide (5.89 mg/mL) and total amino acid (123.06 μmol/mL) levels, indicating enhanced digestibility. Conversely, the CaSO_4_ + MgCl_2_ combination showed delayed proteolysis in electrophoresis analysis. These findings demonstrate that coagulant selection directly modulates tofu’s texture, water retention, and protein bioavailability, with MgCl_2_ favoring digestibility and the hybrid coagulant optimizing physical properties. This provides strategic insights for developing nutritionally enhanced tofu products.

## 1. Introduction

Tofu, a nutrient-dense gel derived from soybeans, is globally esteemed for its health benefits, including high protein content and functional properties [1]. Its quality is largely determined by the coagulation process and the type of coagulant used, both of which directly influence its textural, physicochemical, and sensory attributes [2]. The selection of a coagulant is pivotal in shaping tofu quality, as it impacts not only yield and water retention but also protein digestibility [3,4,5].

Salt-based coagulants, such as calcium sulfate (CaSO_4_) and magnesium chloride (MgCl_2_), are traditionally preferred in tofu production due to their ability to form dense protein networks via ionic interactions with soy proteins [6,7]. These coagulants neutralize the negatively charged carboxyl groups on denatured soy proteins, facilitating hydrophobic interactions and disulfide bond formation. These molecular changes ultimately modulate tofu’s texture, water-holding capacity, and cooking stability [2]. For example, CaSO_4_-coagulated tofu typically exhibits a fine, smooth texture; however, it may suffer from lower water retention and potential bitterness due to residual coagulant [8]. In contrast, MgCl_2_ can enhance soybean flavor retention but may compromise the structural integrity of tofu [9]. These limitations have spurred research into composite coagulants, which aim to integrate the advantages of multiple coagulants to optimize tofu quality.

Recent studies have investigated the use of compound coagulants to address the limitations of single coagulants. For example, Zhao et al. [10] explored mixed-salt systems (e.g., KCl, CaCl_2_, or CaSO_4_ combined with citric acid) and reported improved tofu gel texture and yield, highlighting the potential of coagulant combinations. Similarly, Wang et al. [11] demonstrated that the synergistic use of MgSO_4_, MgCl_2_, and CaSO_4_ improved the gelation properties of soy protein emulsions. These findings underscore the potential of compound coagulants to balance tofu’s texture, water retention, and yield. While advancements have been made in understanding the physicochemical properties of tofu produced with compound coagulants, research on the impact of these salt-based coagulants on protein digestibility remains limited. Protein digestibility is a critical factor in assessing the nutritional quality of tofu, as it determines the bioavailability of essential amino acids and other nutrients [12].

The present study investigates the effects of CaSO_4_, MgCl_2_, and their combination (CaSO_4_ + MgCl_2_) on the physicochemical properties and protein digestibility of tofu. By comparing the water-holding capacity, cooking loss, texture profile, and protein subunit composition of tofu produced with single and compound coagulants, this study aims to clarify how coagulant selection influences tofu quality. Furthermore, an in vitro gastrointestinal digestion model was employed to simulate the digestion process of tofu proteins, analyzing the release of soluble proteins, peptides, and total amino acids. This comprehensive approach aims to (1) elucidate how coagulant type modulates tofu’s physicochemical properties; (2) characterize the impact of coagulants on protein digestibility and bioavailability; and (3) provide valuable guidance for developing nutritionally optimized tofu products.

## 2. Results and Discussion

### 2.1. Analysis of Soluble Protein Content in Soymilk and Soy Whey

The soluble protein content in pre-coagulation soymilk and post-coagulation soy whey was analyzed (Table 1). No significant differences (*p* > 0.05) were observed in soluble protein content among soymilk samples, regardless of coagulant type (Table 1), indicating consistent protein levels prior to coagulation. In contrast, soy whey exhibited significant variations in soluble protein content: CaSO_4_ yielded the highest value, while both MgCl_2_ and the combined coagulant showed significantly lower levels (*p* < 0.05). These results suggest that at an equal mass concentration (0.6% *w*/*v*), CaSO_4_-mediated coagulation leads to greater protein leakage into soy whey. Both CaSO_4_ and MgCl_2_ are salt-based coagulants that facilitate gelation through metal ions (Ca^2+^ or Mg^2+^) binding to proteins, inducing a bridging effect to form a three-dimensional network that entraps water molecules [13]. However, CaSO_4_ released Ca^2+^ ions more slowly, resulting in milder protein interactions and delayed coagulation [6]. This gradual process might allow more soluble proteins to remain unincorporated in the gel matrix, thus increasing their retention in soy whey. Conversely, MgCl_2_ rapidly released Mg^2+^ ions and lowered the pH of the soymilk, accelerating protein denaturation [4] and promoting a compact gel structure. This efficient gelation minimized protein leakage into soy whey. The CaSO_4_ + MgCl_2_ coagulant synergistically combined these effects, enhancing protein aggregation and forming a dense gel network that reduced protein leakage into soy whey compared to CaSO_4_ tofu (*p* < 0.05, Table 1), though not to the level achieved by MgCl_2_ alone (*p* > 0.05).

### 2.2. Electrophoresis Patterns of Soy Protein in Tofu and Soy Whey

**Figure 1:**
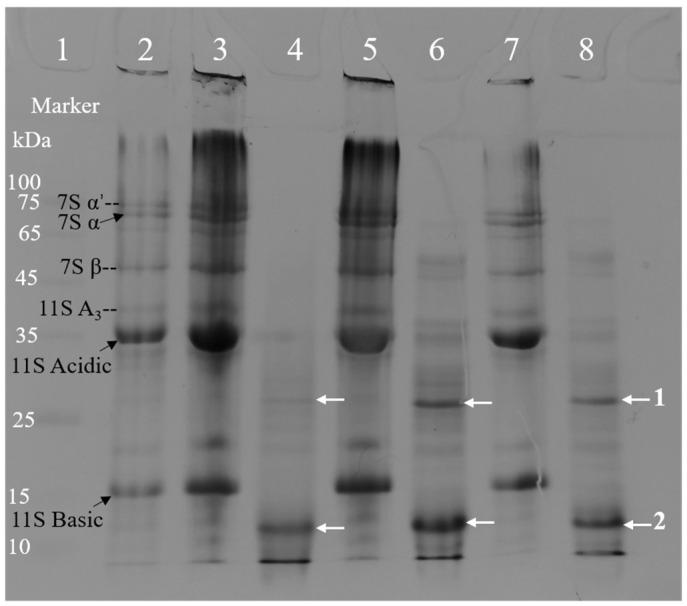
SDS-PAGE profiles of protein marker (lane 1), soymilk (control, lane 2), tofu samples prepared with MgCl_2_ (lane 3), CaSO_4_ (lane 5), and CaSO_4_ + MgCl_2_ (lane 7), with corresponding soy whey fractions from MgCl_2_ tofu (lane 4), CaSO_4_ tofu (lane 6), and composite-coagulant tofu (lane 8). Subunit annotations: 7S α′, 7S α, and 7S β (β-conglycinin); 11S A_3_/Acidic and 11S Basic (glycinin acidic and basic subunits). The arrows indicating 1 and 2 refer to the protein bands detected in soy whey.

Sodium dodecyl sulfate polyacrylamide gel electrophoresis (SDS-PAGE) analysis (Figure 1) shows distinct protein coagulation patterns in tofu and soy whey, depending on the coagulant used. In the control soymilk (lane 2), prominent bands corresponding to β-conglycinin subunits (7S α′ (~75 kDa), 7S α (~68 kDa), 7S β (~50 kDa)) and glycinin subunits (11S A_3_ (~40 kDa), 11S Acidic (~35 kDa), 11S Basic (~15 kDa)) were observed. The tofu samples displayed enhanced retention of these subunits. The MgCl_2_-coagulated tofu (lane 3) showed strong retention of all subunits (7S α′/α/β and 11S Acidic/Basic), indicating the formation of dense gel matrices that trapped aggregated proteins. The intensity difference of the soy protein subunits between lane 3 (MgCl_2_-coagulated tofu) and lane 5 (CaSO_4_-coagulated tofu) was not visually distinct. The hybrid coagulant (CaSO_4_ + MgCl_2_, lane 7) showed similar retention of both 7S and 11S subunits compared with single coagulants.

Corresponding whey samples exhibited an inverse trend in subunit presence. The soy whey released during pressing of MgCl_2_ tofu (lane 4) showed minimal residual bands, while whey from CaSO_4_ tofu (lane 6) retained two strong protein bands (marked by arrows: band 1 (~27 kDa) and band 2 (~12 kDa)), consistent with its highest soluble protein content (Table 1). Conversely, whey from tofu coagulated with the combined coagulant (lane 8) exhibited reduced band retention. These results align with the differing coagulation mechanisms of Mg^2+^ and Ca^2+^, as discussed in Section 2.1.

### 2.3. Water-Holding Capacity and Cooking Loss

**Figure 2:**
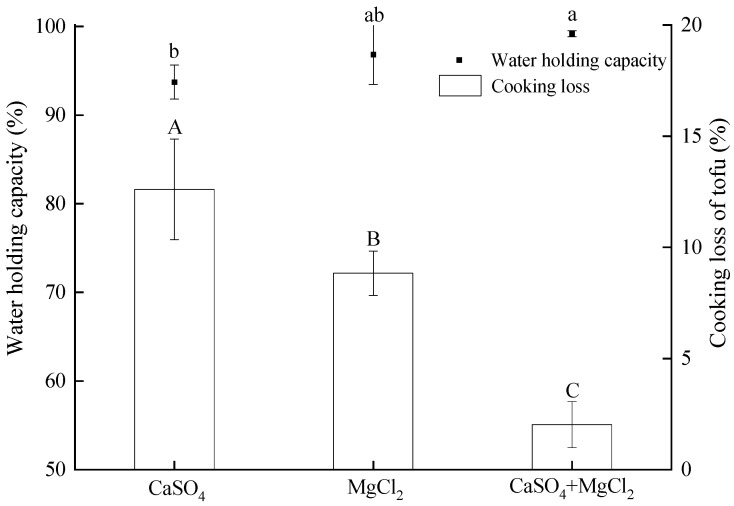
Effects of coagulants on tofu water-holding capacity and cooking loss. Lowercase letters denote significant differences (*p* < 0.05) in water retention. Capital letters denote significant differences (*p* < 0.05) in cooking loss.

Water-holding capacity is a key indicator of tofu texture and internal structural stability. A higher water-holding capacity indicates better moisture retention, whereas a lower water-holding capacity leads to moisture loss, weakening the texture and reducing structural stability [14]. Cooking loss refers to the reduction in food weight due to water expulsion and nutrient leaching during cooking, negatively impacting food quality [15]. Figure 2 illustrates the effects of different coagulants on tofu’s water-holding capacity and cooking loss. The tofu coagulated with CaSO_4_ alone had a water-holding capacity of 93.73 ± 1.92%, while MgCl_2_ coagulation yielded 96.82 ± 3.66%. The CaSO_4_ + MgCl_2_ combination achieved the highest water-holding capacity at 99.16 ± 0.34%. Conversely, cooking loss showed an inverse trend: CaSO_4_ tofu exhibited the highest loss (12.61 ± 2.27%), significantly higher than MgCl_2_ tofu (8.84 ± 1.00%, *p* < 0.05), with the hybrid coagulant resulting in the lowest loss (2.03 ± 1.03%).

The water-holding capacity and cooking loss of tofu are closely linked to coagulant type and the resulting protein network structure. Due to its low solubility, CaSO_4_ induced slow gelation during soymilk coagulation, allowing soy proteins to crosslink in an orderly manner and form a uniform three-dimensional network. This process yielded tofu with weaker mechanical strength and a softer texture [16]. During cooking, the softer structure struggled to retain water, leading to high cooking loss. The combination of CaSO_4_ and MgCl_2_ benefited from the synergistic effects of both coagulants. The slower coagulation kinetics of CaSO_4_ promoted better protein solubility and aggregation, while MgCl_2_ rapidly induced coagulation to refine the protein network. This likely formed a denser and more uniform structure that effectively retained water and minimized cooking loss. Furthermore, the combined coagulants could optimize ionic strength and charge distribution, enhancing protein–protein interactions and the stability of the protein network [11]. The combination of CaSO_4_ and MgCl_2_ outperformed the individual coagulants, providing valuable insights for optimizing tofu production processes.

### 2.4. Textural Properties of Tofu

The texture of tofu is significantly influenced by the choice of coagulant, as it affects the gelation process, microstructure, and mechanical properties of the final product [17]. The textural properties of tofu prepared with different coagulants (CaSO_4_, MgCl_2_ and CaSO_4_ + MgCl_2_) were systematically analyzed, revealing significant variations in hardness and gumminess (Table 2). Specifically, CaSO_4_ tofu exhibited the lowest hardness and gumminess, whereas MgCl_2_ tofu showed intermediate values. The CaSO_4_ + MgCl_2_ combination produced the highest values (hardness, 897.27 ± 56.13 g and gumminess 765.72 ± 47.24), with statistical significance (*p* < 0.05) across all comparisons. The synergistic effect of combining CaSO_4_ and MgCl_2_ likely arises from complementary interactions between calcium and magnesium ions at distinct protein binding sites, further rigidifying the gel structure. In contrast, springiness, cohesiveness, and resilience showed no significant differences across coagulants (*p* > 0.05), indicating that these textural parameters are primarily governed by factors such as moisture content and gel uniformity rather than coagulant type. This aligns with findings by Hu et al. [18], who reported that structural integrity metrics (e.g., cohesiveness) depend more on moisture and gel uniformity than coagulant alone. For instance, glucono-δ-lactone (GDL)-coagulated tofu, despite its low hardness (attributed to uniform protein matrices), also displayed high gumminess due to optimized water retention [9].

Tofu texture preferences are culturally and contextually driven. High-hardness tofu suits frying or stir-frying due to its structural integrity, while medium-hardness tofu is ideal for stews. Low-hardness tofu excels in soups where tenderness is prioritized. Consumer segments also vary: elderly populations may favor softer textures, whereas younger demographics prefer firmer, protein-rich options. Thus, coagulant selection should align with target applications and nutritional demands.

### 2.5. Protein Bioavailability in Tofu Digesta

**Figure 3:**
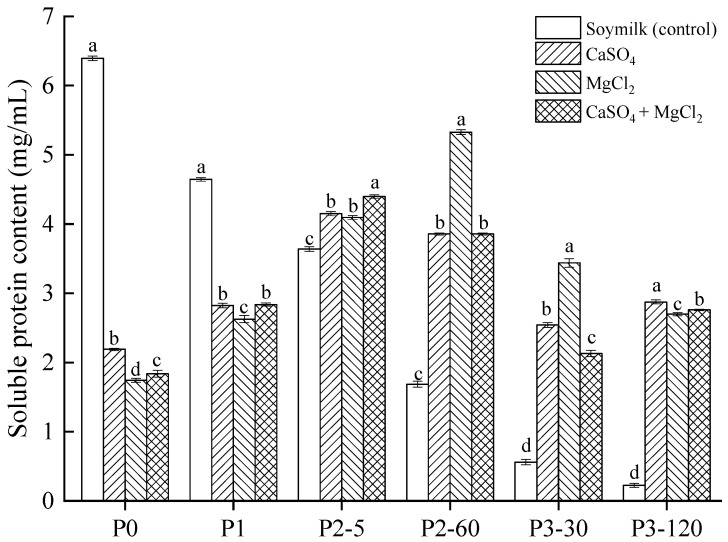
Quantification of soluble proteins during simulated gastrointestinal digestion. Lowercase letters indicate significant variations (*p* < 0.05) within each phase. P0: undigested samples; P1: post-oral digestion; P2-5/P2-60: gastric digestion (5/60 min); P3-30/P3-120: intestinal digestion (30/120 min).

Determining the amount of soluble protein in digesta supernatants is crucial for assessing protein bioavailability [19]. In vitro gastrointestinal simulated digestion of soymilk and tofu samples coagulated with CaSO_4_, MgCl_2_, and CaSO_4_ + MgCl_2_ revealed significant variations in soluble protein content across different digestive phases, as shown in Figure 3. Initially, the soymilk control exhibited the highest soluble protein content at 6.39 mg/mL (P0), which was significantly higher (*p* < 0.05) than the coagulated tofu samples. This aligns with prior research showing that tofu coagulants induce protein denaturation and aggregation, thereby reducing soluble protein content [5].

After oral digestion, soluble protein content in tofu samples increased slightly, primarily due to the mechanical action of chewing, which released partial soluble proteins from the tofu gel. However, α-amylase hydrolysis was limited, yielding no significant increase across the three tofu gel samples. During gastric digestion, soluble protein content rose drastically: at 5 min (P2-5), the MgCl_2_ and CaSO_4_ tofu samples reached 4.09 and 4.15 mg/mL, respectively, while the CaSO_4_ + MgCl_2_ tofu sample showed a significantly higher value of 4.40 mg/mL (*p* < 0.05). This increase stemmed from pepsin disrupting the gel network, releasing more soluble protein from the tofu matrix [20]. After 60 min of gastric digestion (P2-60), the soymilk control decreased to 1.69 mg/mL, while the coagulated tofu samples maintained higher soluble protein content, around 4.0 mg/mL. Notably, the MgCl_2_ tofu sample had significantly higher soluble protein content than the other two tofu samples, reaching 5.33 mg/mL. The structural differences caused by the different coagulants may influence the accessibility of proteins to digestive enzymes, thereby affecting soluble protein content. Murekatete et al. [21] found that CaSO_4_-induced coagulation resulted in a more uniform network structure with a finer, smoother surface compared to MgCl_2_-induced coagulation. Therefore, it can be inferred that soy proteins in CaSO_4_ tofu are less exposed to digestive enzymes, leading to lower soluble protein content. In contrast, MgCl_2_ produced a relatively looser gel structure, allowing proteins to partially retain some solubility. The CaSO_4_ + MgCl_2_ mixed coagulant combined the characteristics of both, creating a gel structure that balanced density and porosity. This structure might partially expose protein molecules to pepsin during gastric digestion, enabling the release of soluble proteins.

However, as digestion progressed into the intestinal phase (P3-30 and P3-120), soluble protein content decreased relative to the gastric phase, likely due to further proteolysis into smaller peptides and amino acids that were undetectable by the Bradford assay [15,22]. The decline in soluble proteins during the intestinal phases reflects both the structural limitations to enzyme access and progressive proteolysis into peptides and amino acids; the latter are further quantified in Section 2.6. These findings emphasize the interplay between coagulant type and protein bioavailability, with implications for nutritional outcomes.

### 2.6. Peptide and Total Amino Acid Contents Analysis

**Figure 4:**
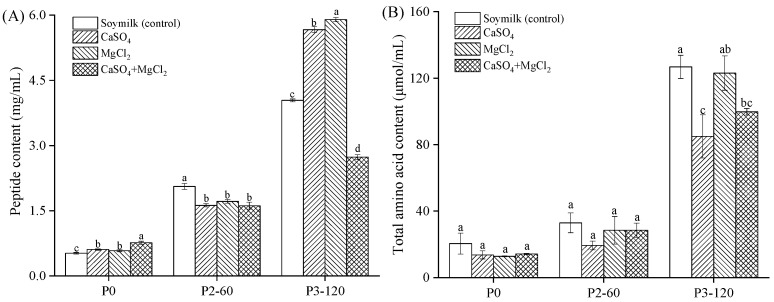
Hydrolysis products during digestion: (**A**) peptide and (**B**) amino acid levels. Significant differences (*p* < 0.05) within phases are marked by lowercase letters. P0: undigested samples; P2-60: gastric digestion of 60 min; P3-120: intestinal digestion of 120 min.

Different coagulants affect the aggregation of soybean proteins and the structure of tofu gel networks, thereby influencing its digestive characteristics [23]. During gastrointestinal digestion, proteins are hydrolyzed by proteases, such as pepsin and pancreatin, into peptides and amino acids. Peptides, as intermediate products of protein hydrolysis, provide insights into the extent and dynamics of protein digestion. The in vitro gastrointestinal simulated digestion of soymilk and tofu samples coagulated with CaSO_4_, MgCl_2_, and CaSO_4_ + MgCl_2_ revealed significant differences in peptide and amino acid contents across various digestive phases, as shown in Figure 4.

Initially (P0), the peptide (Figure 4A) and amino acid (Figure 4B) contents were relatively low. The soymilk control exhibited 0.52 mg/mL and 20.47 μmol/mL, respectively, while the coagulated tofu samples ranged from 0.61 to 0.76 mg/mL and 12.79 to 14.21 μmol/mL (P0). After gastric digestion (P2-60), peptide content in all the samples slightly increased, reaching approximately 1.6 mg/mL for the CaSO_4_ and CaSO_4_ + MgCl_2_ tofu samples and 1.8 mg/mL for the MgCl_2_ tofu sample, with no significant differences between tofu samples (*p* > 0.05). Amino acid content also slightly increased, reaching 19.29–28.47 μmol/mL in the tofu samples, with no significant difference compared with the soymilk control (32.94 μmol/mL).

Notably, during intestinal digestion, the peptide content peaked at 120 min (P3-120), with the MgCl_2_ tofu sample showing the highest value of 5.89 mg/mL, significantly higher than the CaSO_4_ tofu (5.67 mg/mL), soymilk (4.04 mg/mL), and CaSO_4_ + MgCl_2_ tofu (2.73 mg/mL) samples. Similarly, at the end of intestinal digestion (P3-120), the MgCl_2_ tofu exhibited the highest total amino acid content (123.06 μmol/mL), significantly higher than the amino acid content in the CaSO_4_ tofu (84.94 μmol/mL, *p* < 0.05). The amino acid content in the mixed coagulant tofu (99.76 μmol/mL) was intermediate, with no significant difference compared to the other two groups (*p* > 0.05). These findings suggest that MgCl_2_ coagulant enhanced peptide release during digestion, likely due to its impact on tofu gel structure and protein solubility, making it more susceptible to enzymatic breakdown. Hu et al. [18] compared the effects of three coagulants (MgCl_2_, CaSO_4_, GDL) on tofu structure and protein digestibility, noting that GDL tofu exhibited higher water retention and lower hardness, with its protein digestibility likely related to its structure. Liu et al. [15] explored the effects of CaSO_4_ and transglutaminase (TGase) combined treatment on tofu quality and digestibility, finding that TGase pre-crosslinking significantly improved tofu’s physicochemical properties and protein digestibility, suggesting that gel network modification can influence digestion. Lou et al. [20] used an artificial gastric digestive system to study the in vitro digestion of different tofu types (using GDL and CaSO_4_ as coagulants), observing that protein hydrolysis and amino acid accumulation in GDL tofu were faster than in CaSO_4_ tofu. This further supports the impact of coagulants on tofu digestibility. The time-dependent increase in peptide content emphasizes the cumulative nature of the digestive process and the role of digestive enzymes in protein hydrolysis. These results provide insights into the optimization of tofu processing for enhanced nutritional value and digestibility.

### 2.7. SDS-PAGE Analysis of the Tofu Digesta

**Figure 5:**
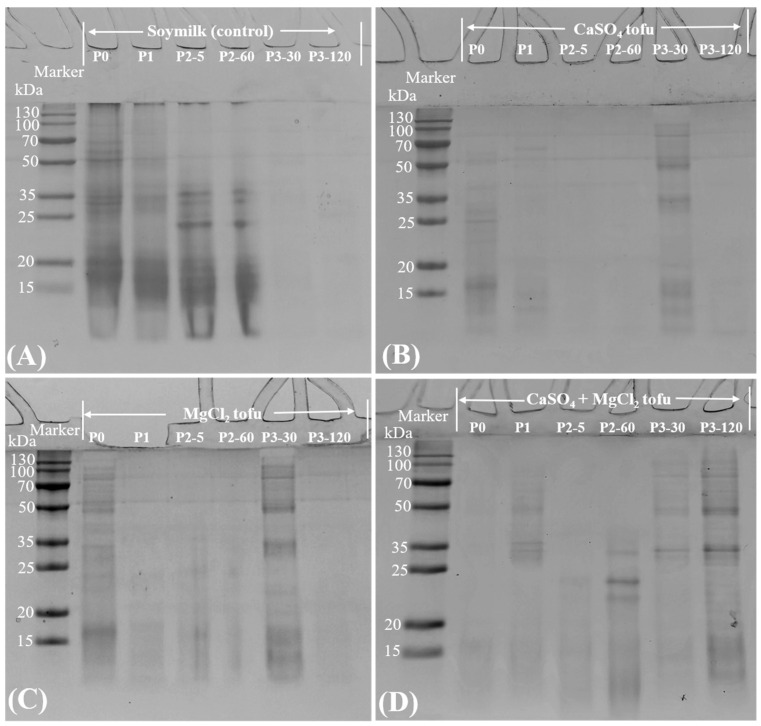
SDS-PAGE analysis of digestion stages: (**A**) Soymilk (control), (**B**) CaSO_4_-, (**C**) MgCl_2_-, and (**D**) CaSO_4_ + MgCl_2_-coagulated tofu. Phases: pre-digestion (P0), post-oral (P1), gastric phase (P2-5, P2-60), and intestinal phase (P3-30, P3-120).

SDS-PAGE analysis revealed distinct proteolytic patterns in the tofu samples coagulated with CaSO_4_, MgCl_2_, and their combination (CaSO_4_ + MgCl_2_) during simulated gastrointestinal digestion, as illustrated in Figure 5. In the control (soymilk, Figure 5A), undigested samples (P0) exhibited prominent bands between 15~70 kDa, corresponding to β-conglycinin (7S) and glycinin (11S), consistent with typical soy protein profiles [24]. Buccal digestion (P1) showed minimal changes as expected, due to the absence of proteases. The gastric phases (P2-5 and P2-60) displayed progressive hydrolysis, with high-molecular weight (MW) bands diminishing and lower-MW fragments (20~35 kDa) emerging, indicative of pepsin activity [25]. Intestinal digestion (P3-30 and P3-120) nearly degraded residual proteins completely.

CaSO_4_- (Figure 5B) and MgCl_2_-coagulated tofu (Figure 5C) exhibited similar proteolytic profiles, with high-MW bands disappearing during the gastric digestion phase and new fragments (~35 and 50 kDa) emerging at P3-30. The CaSO_4_ + MgCl_2_ tofu sample (Figure 5D) demonstrated delayed proteolysis, with persistent low-MW bands (20~35 kDa) at P2-60 and residual fragments (35~50 kDa) remaining at P3-30 and P3-120. These results emphasize that mixed salt coagulants critically modulate tofu protein degradation, with implications for designing functional foods that target controlled nutrient release.

## 3. Conclusions

This study systematically investigated the effects of CaSO_4_, MgCl_2_, and their combination on the physicochemical properties and protein digestibility of tofu, providing critical insights into salt-based coagulant-driven optimization of tofu quality and nutritional value. Key findings revealed that the hybrid coagulant (CaSO_4_ + MgCl_2_) synergistically enhanced tofu’s structural integrity, water-holding capacity, and cooking stability, outperforming single coagulants. The combination achieved a denser, more uniform protein network by balancing the rapid Mg^2+^-induced coagulation and the slower Ca^2+^-mediated gelation, thereby minimizing protein leakage into soy whey and improving textural hardness and gumminess. Notably, coagulant type significantly influenced protein digestibility. While MgCl_2_-coagulated tofu exhibited higher soluble protein and peptide release during gastric and intestinal phases, suggesting enhanced enzymatic accessibility due to its looser gel structure, the hybrid coagulant demonstrated intermediate digestibility patterns. SDS-PAGE analysis further highlighted distinct proteolytic profiles, with the hybrid coagulant delaying protein degradation and retaining residual fragments, potentially enabling controlled nutrient release. These findings underscore that coagulant selection directly modulates tofu’s protein bioavailability, balancing structural robustness with digestibility.

However, limitations include the use of an in vitro digestion model, which may not fully replicate human physiological conditions, and the lack of optimization for coagulant ratios or concentrations. Future studies should validate these findings through in vivo trials, explore synergistic effects with novel coagulants (e.g., enzymatic or acid-based systems), and assess sensory attributes to align technical improvements with consumer preferences. This work advances the scientific understanding of tofu gelation mechanisms and provides actionable guidelines for developing high-quality, nutritionally optimized plant-based protein products.

## 4. Materials and Methods

### 4.1. Materials

Soybeans were purchased from a local supermarket in Changshu, China. MgCl_2_ was obtained from Lianyungang Rifeng Calcium & Magnesium Co., Ltd., Lianyungang, China. CaSO_4_ was sourced from Angel Yeast Co., Ltd., Yichang, China. Bovine serum albumin (BSA) and Coomassie Brilliant Blue G250 were purchased from Phygene Biotechnology Co., Ltd., Fuzhou, China. Sodium dodecyl sulfate (SDS), borax, and o-phthalaldehyde (OPA) were sourced from Yien Chemical Technology Co., Ltd., Shanghai, China. The SDS-PAGE gel rapid preparation kit and protein marker (15~130 kDa) were obtained from Labgic Technology Co., Ltd., Hefei, China. Pepsin (P8390), α-amylase (G8290), pancreatin powder (P7340), porcine bile salts (G8310), β-mercaptoethanol, and amino acid content assay kits (BC1570) were purchased from Solarbio Science & Technology Co., Ltd., Beijing, China. All other reagents, unless specified otherwise, were of analytical grade.

### 4.2. Preparation of Soymilk and Tofu

Three batches of plump soybeans (200 g each) were soaked overnight in distilled water (600 mL), then ground into soymilk using a commercial blender (Model L18-Y915S, Joyoung Co., Ltd., Hangzhou, China) at a ratio of 1:6 (*w*/*v*, dry soybeans to water). The slurry was filtered to remove okara, and the raw soymilk was boiled for 5 min with continuous stirring to prevent scorching. After heating, the cooked soymilk was quickly transferred to an 85 °C water bath for temperature maintenance. Subsequently, a 10% (*w*/*v*) MgCl_2_ solution was added to achieve a final concentration of 0.6% (*w*/*v*) in the soymilk to prepare MgCl_2_-coagulated tofu; a 14.5% (*w*/*v*) CaSO_4_ suspension was added to achieve a final concentration of 0.6% (*w*/*v*) in the soymilk to prepare CaSO_4_-coagulated tofu; and a mixture of 10% (*w*/*v*) MgCl_2_ solution and 14.5% (*w*/*v*) CaSO_4_ suspension was added to achieve final concentrations of 0.3% (*w*/*v*) each in the soymilk to prepare CaSO_4_ + MgCl_2_-coagulated tofu, according to the method described by Hu et al. [18], with some modifications. All soymilk treated with the coagulants mentioned above was maintained at 85 °C for 30 min. The curds were pressed into tofu under 20 g cm^−2^ pressure for 50 min, with each coagulant used in triplicate.

### 4.3. Water-Holding Capacity Measurement

Water-holding capacity was measured using high-speed centrifugation, as modified from Cao et al. [26]. Tofu samples were cut into strips of 2.0 cm × 1.0 cm × 1.0 cm and centrifuged at 5000× *g* for 10 min using a micro high-speed centrifuge (Model MC-15K, Hangzhou Youning Instrument Co., Ltd., Hangzhou, China). The ratio of the tofu mass after centrifugation to the mass before centrifugation was calculated as the water-holding capacity (%) using the formula provided below:

Water-holding capacity (%) = [(M2 − M0/(M1 − M0)] × 100(1)

where M0 = mass of empty centrifuge tube (g); M1 = total mass of centrifuge tube and tofu before centrifugation (g); M2 = total mass of centrifuge tube and tofu after centrifugation (g). The result was reported as the mean value of four replicates.

### 4.4. Determination of Cooking Loss

Following the method of Lan et al. [27], tofu samples prepared with different coagulants were cut into cubes (2.0 cm × 2.0 cm × 1.0 cm) and accurately weighed, and the initial mass (W1) was recorded. The tofu cubes were then placed in boiling water and cooked for 5 min. After cooking, the samples were quickly transferred to a 40-mesh sieve for filtration. After the samples cooled to room temperature, their final mass (W2) was measured. Cooking loss was calculated using the following formula:

Cooking loss (%) = [(W1 − W2)/W1] × 100(2)

where W1 represents the mass of the tofu sample before cooking (g), and W2 represents the mass of the tofu sample after cooking and filtration (g). The analysis was conducted in sextuplicate.

### 4.5. Texture Profile Analysis (TPA) of Tofu

TPA was conducted to assess the gel strength of tofu using a TA.X2i texture analyzer (Godalming, Surrey, UK) equipped with a TA35 probe, following a previously established protocol with minor modifications [28]. The tofu samples were compressed using a cylindrical probe with a diameter of 3.5 cm to achieve 30% deformation. Four replicates were conducted for each sample. Texture parameter (hardness) is reported in grams-force (g), consistent with standard TPA reporting conventions.

### 4.6. In Vitro Gastrointestinal Digestion of Tofu

Following the model of Hui et al. [29], tofu prepared with different coagulants underwent a three-sequential phase digestion process (oral, gastric, and intestinal) to simulate human digestion. Briefly, 60 g of ground sample was mixed with 24 mL α-amylase solution (0.2 mg/mL in 20 mM phosphate buffer, pH 7.0) and shaken at 55 r/min (37 °C, 3 min) for oral digestion. A 7 g aliquot was collected as P1. The pH was then adjusted to 2.0 with 4 M HCl, followed by the addition of 33 mL pepsin solution (3.2 mg/mL in 0.1 M HCl). Gastric digestion proceeded at 55 r/min (37 °C, 60 min), with 10 g samples taken at 5 min (P2-5) and 60 min (P2-60). For intestinal digestion, the pH was adjusted to 7.0 with 4 M NaOH; 18 mL pancreatin and 18 mL bile (0.4 mg/mL in phosphate buffer, pH 7.0) were added. Digestion continued at 150 r/min (37 °C, 120 min), with 14 g samples collected at 30 min (P3-30) and 120 min (P3-120). After each phase, enzymes were heat-inactivated (100 °C, 5 min), and supernatants were collected via centrifugation (10,000× *g*, 15 min).

### 4.7. Determination of Soluble Protein Content

Soluble protein content in soymilk, soy whey from tofu manufacturing with different coagulants, and digesta samples were quantified using the Bradford assay [30]. Samples were appropriately diluted, and 1 mL of the diluted solution was mixed with 5 mL of Coomassie Brilliant Blue G-250 solution, incubated for 5 min. Absorbance at 595 nm was measured using a ultraviolet–visible spectrophotometer (Model 754 PC, Jinghua Instruments Co., Ltd., Shanghai, China). A standard curve was generated with bovine serum albumin (BSA), and the results were expressed as a BSA-equivalent concentration (μg/mL). Distilled water substituted the sample as a blank control. Each sample was analyzed in triplicate.

### 4.8. Determination of Low Peptide Content

Low peptide content in tofu digesta (P0, P2-60, P3-120) was quantified following Rui et al. [28]. Samples were filtered through a 10 kDa molecular weight cutoff membrane (Millipore, Billerica, MA, USA) by centrifugation (10,000× *g*, 10 min). Filtrate aliquots (50 µL) were mixed with 2 mL reaction solution containing 100 mM borax (25 mL), 20% (*w*/*v*) SDS (2.5 mL), OPA (40 mg dissolved in 1 mL of methanol), and β-mercaptoethanol (100 µL), with the final volume adjusted to 50 mL. The results were expressed as casein tryptone equivalents, with each sample analyzed in triplicate.

### 4.9. Total Amino Acid Content Analysis

Total amino acid content in tofu digesta (P0, P2-60, P3-120) was measured using the ninhydrin colorimetric method according to the instructions in the Solarbio amino acid content detection kit (BC1570) [4]. First, 0.5 mL of each sample was mixed with an equal volume of reagent 1 and heated in a boiling water bath for 15 min. After cooling, the mixture was centrifuged at 10,000× *g* for 10 min at 4 °C, and the supernatant was collected. Then, 50 µL of the supernatant was mixed with 500 µL of reagent 2, 500 µL of reagent 3, and 50 µL of reagent 4, and the mixture was heated in a boiling water bath for 15 min. After cooling, the mixture was centrifuged again, and the supernatant was measured for absorbance at 570 nm using an ultraviolet–visible spectrophotometer (754 PC, Jinghua Instruments Co., Ltd., Shanghai, China). Each sample was tested in triplicate.

### 4.10. SDS-PAGE

Protein molecular weights in the soymilk, soy whey, tofu, and digestion-stage samples were determined by SDS-PAGE. To each well, 20 µL of protein sample was mixed with 5 µL of sample buffer (Qianzhusong Biological Technology Co., Ltd., Zhenjiang, China) and boiled for 5 min. Then, 10 µL of the boiled sample was loaded onto a 4% stacking gel and a 12% separating gel for electrophoresis. Protein markers (15–130 kDa) were used as references. The gel was run at a constant voltage of 60 V for 1 h, then increased to 120 V for 90 min. Gels were stained with a solution of 40% ethanol, 10% acetic acid, and Coomassie Brilliant Blue R-250 and destained until the protein bands were clearly visible. The bands were analyzed for intensity using Quantity One software (version 4.6.2, Bio-Rad, Laboratories, Inc., Hercules, CA, USA).

### 4.11. Statistical Analysis

All experiments were conducted a minimum of three times, and data are presented as the mean ± standard deviation. Statistical analyses were performed using SPSS version 16.0 (SPSS Inc., Chicago, IL, USA). Analysis of variance (ANOVA), followed by Duncan’s multiple range test, was employed to identify significant differences between the means, with a significance threshold set at *p* < 0.05.

## Figures and Tables

**Table 1:** Soluble protein concentrations in soymilk and soy whey obtained during tofu preparation with distinct coagulants.

Type of Coagulant	Sample Type	Soluble Protein Concentration (mg/mL) ^1^
CaSO_4_	Soymilk Soy whey	35.07 ± 0.18 ^A^ 3.32 ± 0.23 ^a^
MgCl_2_	Soymilk Soy whey	34.44 ± 0.39 ^A^ 1.92 ± 0.02 ^b^
CaSO_4_ + MgCl_2_	Soymilk Soy whey	34.51 ± 0.27 ^A^ 2.11 ± 0.08 ^b^

^1^ Capital letters (A) indicate no significant differences among soymilk groups (*p* > 0.05); lowercase letters (a and b) indicate significant differences among soy whey groups (*p* < 0.05).

**Table 2:** Textural properties of tofu coagulated with different coagulants.

Type of Coagulant	Hardness (g)	Springiness	Cohesiveness	Gumminess	Resilience ^1^
CaSO_4_	214.33 ± 15.88 ^c^	0.98 ± 0.00 ^a^	0.84 ± 0.03 ^a^	180.17 ± 11.65 ^c^	0.54 ± 0.03 ^a^
MgCl_2_	645.41 ± 57.75 ^b^	0.99 ± 0.00 ^a^	0.85 ± 0.02 ^a^	551.59 ± 51.60 ^b^	0.57 ± 0.03 ^a^
CaSO_4_ + MgCl_2_	897.27 ± 56.13 ^a^	0.99 ± 0.00 ^a^	0.85 ± 0.01 ^a^	765.72 ± 47.24 ^a^	0.56 ± 0.00 ^a^

^1^ Lowercase letters that differ within the same column indicate significant difference (*p* < 0.05).

## Data Availability

The data presented in this study are available on request from the corresponding author due to reasonable request.
